# Pulmonary manifestations of POEM syndrome: a retrospective analysis of 282 cases

**DOI:** 10.1186/s12890-023-02741-9

**Published:** 2023-11-23

**Authors:** Yilin Huang, Yang Luo, Huan Hou, Jinming Gao

**Affiliations:** 1 Department of Pulmonary and Critical Care Medicine, Peking Union Medical College Hospital, Peking Union Medical College & Chinese Academy of Medical Sciences, Beijing, China; 2 Peking University HuiLongGuan Clinical Medical School, Beijing HuiLongGuan Hospital, Beijing, 100096 People’s Republic of China

**Keywords:** POEMS syndrome, Pulmonary hypertension, Pleural effusion, Pulmonary function test, Plasma cell disorder

## Abstract

**Background:**

Polyneuropathy, organomegaly, endocrinopathy, monoclonal gammopathy, and skin changes (POEMS) syndrome is a rare multisystemic clonal plasma cell disorder. Pulmonary involvement is frequently found in patients with POEMS syndrome, manifesting various clinical features. Therefore, to improve diagnostic accuracy and provide treatment strategies, a comprehensive analysis of pulmonary manifestations of POEMS syndrome is needed.

**Methods:**

This retrospective study included patients with POEMS syndrome at Peking Union Medical College Hospital, a major referral medical center in China, between June 1, 2013, and June 1, 2023. Demographic data, laboratory findings, pulmonary function test results, echocardiograms, and chest imaging data were extracted. Continuous variables were compared using the t-test or Mann–Whitney method. Pearson’s chi-square test or Fisher’s exact test was conducted to compare categorical data.

**Results:**

Overall, 282 individuals diagnosed with POEMS syndrome were included in this study, of which 56% were male with an average age of 48.7 years. Respiratory symptoms were found in 40.1% of the patients, with dyspnea as the most common symptom (34.4%). Chest computed tomography and echocardiography findings showed that 56.4% of patients exhibited pleural effusion, 62.8% displayed mediastinal or hilar lymphadenopathy, 46.5% presented pleural thickening, 27.3% demonstrated bone lesions of the ribs or thoracic vertebra, 7.8% showed lung interstitial abnormalities, and 35.5% had pulmonary hypertension. Decreased diffuse capacity and restrictive ventilatory patterns were identified in 85.2% (115 cases) and 47.4% (64 cases) of patients, respectively. Patients with respiratory symptoms exhibited higher declined lung function measures than those having no respiratory symptoms. High-risk patients with poor prognosis showed more pulmonary function abnormalities.

**Conclusion:**

Abnormalities in pulmonary manifestations constitute the significant features of POEMS syndrome. Several patients with POEMS syndrome presented with respiratory symptoms at the initial evaluation. These findings underscore the importance of early identification and accurate diagnosis of POEMS syndrome by clinicians, particularly in cases involving lung and multisystem.

## Background

Polyneuropathy, organomegaly, endocrinopathy, monoclonal gammopathy, and skin changes (POEMS) syndrome, known less frequently as Crow-Fukase syndrome or Takatsuki syndrome, is a rare multisystemic clonal plasma cell disorder [1]. However, not all patients exhibit all aspects, and the acronym does not encompass all manifestations of this syndrome. Atypical features were noted in patients with POEMS syndrome, such as extravascular volume overload (manifesting as peripheral edema, ascites, and pleural effusions), predisposition towards thrombosis, diarrhea, and elevated vascular endothelial growth factor (VEGF) levels [2]. A Japanese national survey on POEMS syndrome conducted in 2003 reported a prevalence of 0.3 cases per 100,000 individuals [3]. In China, POEMS syndrome has increasingly received attention [4].

Although the pathogenesis of POEMS syndrome is poorly understood, VEGF is widely considered to be responsible for the various manifestations, and has been used as a reliable biomarker for diagnosis and monitoring [5, 6]. VEGF is a cytokine synthesized by plasma cells, and its receptors are primarily located in endothelial cells, chondrocytes, hematopoietic stem cells, and monocytes. It increases vascular permeability, leading to pleural effusion, ascites, and peripheral edema [1]. Proinflammatory cytokines, such as interleukin 6 (IL-6), IL-1, and tumor necrosis factor (TNF)-α, are also considered to be involved in the pathogenesis, which may underlie the limited efficacy of VEGF inhibitors [7, 8]. Recently, the monoclonal immunoglobulin light chain was reported to play an important role in disease pathogenesis [9].

POEMS syndrome is a multisystemic and chronic disorder that clinicians frequently overlook at the early stages of diseases. Therefore, a correct diagnosis should be established based on the combination of various multisystem symptoms and laboratory findings. Misdiagnosis can lead to delayed treatment and poor clinical outcomes, with approximately 31.9% of patients inappropriately treated with glucocorticoids before a diagnosis of POEMS syndrome [4]. Although lung manifestations are excluded from the diagnostic criteria, several case reports and observational studies have demonstrated the relationship between POEMS syndrome and pulmonary diseases [10–14]. The respiratory physicians’ lack of awareness of this disease, and sole focus on specific conditions while disregarding other abnormal manifestations, leads to misdiagnosis and, ultimately, poor prognosis. Accordingly, a comprehensive analysis of the respiratory manifestations of POEMS syndrome is urgently required. To evaluate the pulmonary involvement of POEMS syndrome, we collected demographic and laboratory data to better understand the clinical presentations of this syndrome and improve the efficiency of diagnosis.

## Methods

### Patients and data collection

This study was reviewed and approved by the ethics committee of Peking Union Medical College Hospital (approval number I-23ZM0018). A retrospective computerized database search of medical records was conducted to identify all patients with POEMS syndrome between June 1, 2013, and June 1, 2023. According to the criteria updated in 2021, a diagnosis of POEMS syndrome was confirmed when both the mandatory major criteria (polyradiculoneuropathy and monoclonal plasma proliferation), one of the three other major criteria (Castleman disease, elevated VEGF levels, and sclerotic bone lesions), and one of the six minor criteria (organomegaly, extravascular volume overload, endocrinopathy, skin changes, papilledema, and thrombocytosis/polycythemia) were present [2]. Some patients without monoclonal gammopathy or polyneuropathy were diagnosed with atypical POEMS syndrome variants by hematologists owing to other suggestive clinical features.

We collected demographic information, including sex, age, smoking status, history of alcohol consumption, comorbidities, duration of diagnosis, and length of hospital stay. Clinical signs of POEMS syndrome and respiratory symptoms, such as dyspnea, chest tightness, orthopnea, and cough, were documented at the initial admission. The following laboratory test results were obtained: levels of albumin, lactate dehydrogenase (LDH), IL-6, TNF-α, complement component 3 (C3), C4, β-isomerized C-terminal telopeptides, VEGF, kappa light chain (KAP), and lambda light chain (LAM). Chest computed tomography (CT) scans or radiography reports were assessed by a clinical radiologist. Imaging evaluation included that for pleural effusion, lymphadenopathy, pleural thickening, bone lesions, and interstitial lung disease. Abnormal echocardiograms during diagnosis were recorded, including those for pulmonary hypertension, heart enlargement, pericardial effusion, systolic dysfunction, and diastolic dysfunction. Systolic pulmonary artery pressure (sPAP) estimated using echocardiography was categorized into the following three grades according to its severity: 35 ≤ mild sPAP ≤49 mmHg, 50 ≤ moderate sPAP ≤69 mmHg, and severe sPAP ≥70 mmHg. Furthermore, pulmonary function test (PFT) data were abstracted to analyze the forced expiratory volume in one second (FEV_1_), FEV_1_ percentages of predicted value (FEV_1_%), forced vital capacity (FVC), FVC percentage of predicted value (FVC%), diffusing capacity of the lung for carbon monoxide percentages of predicted value (DLCO%), and total lung capacity percentages of predicted value (TLC%). Obstructive lung dysfunction was defined as FEV_1_/FVC < 70%, and restrictive dysfunction as TLC% < 80 and FEV_1_/FVC ≥70%. DLCO% < 80%, after adjustment for hemoglobin, was considered as diffuse capability dysfunction. Risk stratification of patients was calculated according to the predictive model developed by Wang et al., including age > 50 years, pulmonary hypertension, pleural effusion, and estimated glomerular filtration rate of < 30 ml/min/1.73 m^2^ [15].

### Statistical analyses

Data were analyzed using IBM SPSS Statistics for Windows, version 25.0 (IBM Corp., Armonk, NY, USA). Categorical data are presented as percentages, whereas continuous variables are expressed as mean ± standard deviation (for normally distributed data) or median (interquartile range) (for non-normally distributed data). Categorical data were compared using Pearson’s chi-square test or Fisher’s exact test, while continuous variables were compared using t-tests or the Mann–Whitney method. Multivariate analysis was performed using logistic regression. Statistical significance was set at *P* < 0.05.

## Results

### Demographics and laboratory test results

Overall, 282 patients were included in this study. The mean age was 48.7 ± 10.8 years, and 56.0% were male. Table 1 shows the baseline characteristics of patients with POEMS syndrome. The reported respiratory symptoms were dyspnea (34.4%), chest tightness (21.6%), orthopnea (16.3%), and cough (5.3%). Extrapulmonary symptoms included polyneuropathy (93.6%), organomegaly (92.2%), and endocrinopathy (87.2%). Hypertension was the most common comorbidity (27.7%). The median duration of diagnosis since the occurrence of symptoms was 1.3 years, with a median hospital stay of 10 days. Laboratory tests revealed elevated levels of serum IL-6 (median value 6.2 pg/mL [normal, < 5.9 pg/mL]) and TNF-α (median value 13.9 [normal, < 8.1 pg/mL]). A total of 85.5% of patients exhibited serum VEGF levels exceeding 1200 pg/mL.

**Table 1:** Clinical and laboratory characteristics of patients with POEMS syndrome

	ALL	male	female
N, (%)	282 (100.0)	158 (56.0)	124 (44.0)
Age, years	48.7 ± 10.8	48.7 ± 11.6	48.6 ± 9.8
Smoking, N (%)	71 (25.2)	70 (44.3)	1 (0.8)
Alcohol, N (%)	40 (14.2)	39 (24.7)	1 (0.8)
Respiratory symptoms			
Dyspnea, N (%)	97 (34.4)	29 (18.4)	32 (25.8)
Chest tightness, N (%)	61 (21.6)	47 (29.7)	50 (40.3)
Orthopnea, N (%)	46 (16.3)	15 (9.5)	31 (25)
Cough, N (%)	15 (5.3)	8 (5.1)	7 (5.6)
Comorbidities			
Hypertension, N (%)	78 (27.7)	43 (27.2)	35 (28.2)
Cardiac disease, N (%)	14 (5)	12 (7.6)	2 (1.6)
Diabetes, N (%)	37 (13.1)	25 (15.8)	12 (9.7)
Cancer, N (%)	5 (1.8)	2 (1.3)	3 (2.4)
POEMS manifestations			
Polyneuropathy, N (%)	264 (93.6)	148 (93.7)	116 (93.5)
Organomegaly, N (%)	260 (92.2)	141 (89.2)	119 (96)
Endocrinopathy, N (%)	246 (87.2)	145 (91.8)	101 (81.5)
Monoclonal gammopathy, N (%)	245 (86.9)	135 (85.4)	110 (88.7)
Skin changes, N%	245 (86.9)	138 (87.3)	107 (86.3)
Bone lesions, N (%)	158 (56)	100 (63.3)	58 (46.8)
Peripheral oedema, N (%)	218 (77.3)	117 (74.1)	101 (81.5)
Papilledema, N (%)	100 (35.5)	49 (31)	51 (41.1)
Castleman disease, N (%)	41 (14.5)	14 (8.9)	27 (21.8)
Laboratory test results			
Albumin, g/L	35.1 ± 5.8	35.2 ± 6.0	35.0 ± 5.7
LDH, U/L	133.5 ± 43.8	139.5 ± 50.7	125.9 ± 31.7
IL-6, pg/mL	6.2 (3.5–13.1)	6.2 (3.4–11.8)	6.65 (3.6–13.8)
TNF-α, pg/mL	13.9 (10.4–20.6)	15.4 (11.1–21.1)	11.8 (9.6–20.5)
C3, g/L	0.9 ± 0.2	0.9 ± 0.2	1.0 ± 0.2
C4, g/L	0.2 ± 0.1	0.2 ± 0.1	0.2 ± 0.1
β-CTX, ng/ml	1.2 (0.8–1.7)	1.2 (0.8–1.8)	1.1 (0.8–1.6)
KAP, %	7 (2.5)	4 (2.5)	3 (2.4)
LAM, %	235 (83.3)	130 (82.3)	105 (84.7)
VEGF > 1200 pg/mL, %	241 (85.5)	133 (84.2)	108 (87.1)
Duration of diagnosis, years	1.3 (0.6–2)	1.2 (0.6–2.0)	1.3 (0.6–2)
Length of stay, days	10.0 (4.0–21.3)	9.0 (4.0–21.0)	12.5 (5.0–23.0)

*LDH *lactatedehydrogenase, *IL-6* interleukin 6, *TNF-α* tumor necrosis factor α, *C3* complement component 3, *C4* complement component 4, *β-CTX* β-type I collagen carboxyterminalpeptide, *KAP* Kappa light chain, *LAM* Lambda light chain, *VEGF* vascular endothelial growth factor

**Fig. 1:**
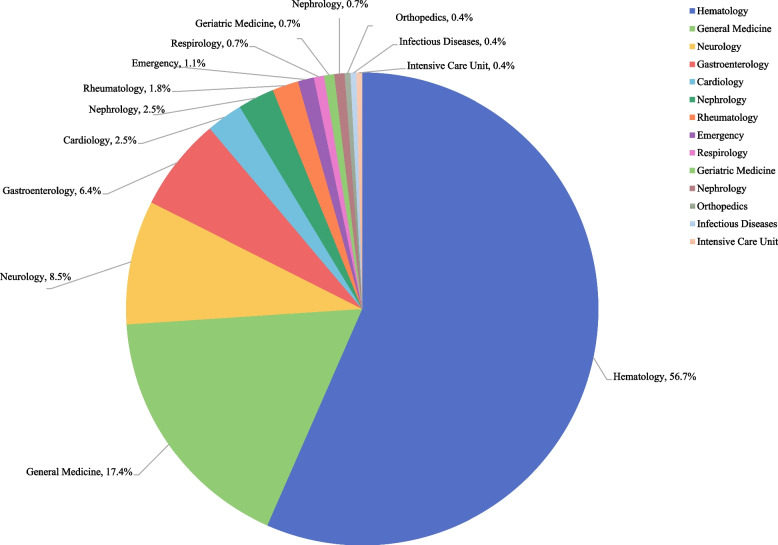
Number of diagnosed patients in different departments

A spectrum of diagnostic patterns for POEMS syndrome was found across departments (Fig. 1). Hematology (160 cases, 56.7%), general medicine (49 cases, 17.4%), and neurology (24 cases, 8.5%) reported relatively higher diagnosis rates, while other departments, including respirology (2 cases, 0.7%), reported fewer cases.

### Pulmonary manifestations

**Fig. 2:**
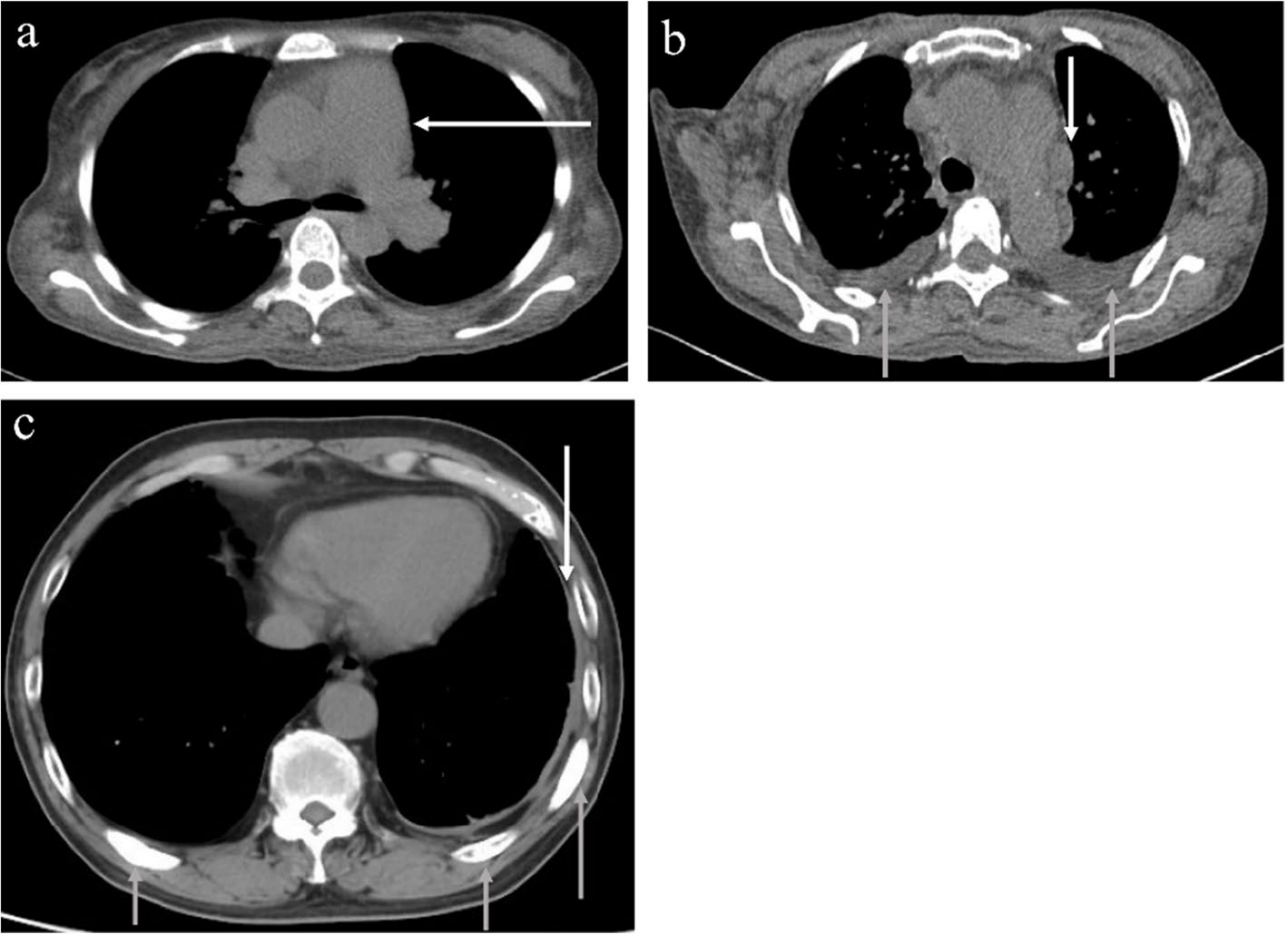
Chest CT of patients with POEMS syndrome. **a** A 39-year-old woman with pulmonary artery widening (white arrows). **b** A 53-year-old woman with an enlarged lymph node (white arrows), and bilateral pleural effusion (gray arrows). **c** A 67-year-old man with pleural thickening (white arrows), and bone lesions (gray arrows)

All patients underwent chest thin-section CT, radiography, and echocardiography during the diagnosis of POEMS syndrome. Overall, 262 patients displayed abnormal pulmonary manifestations (Fig. 2), of whom 159 (56.4%) exhibited pleural effusion, 177 (62.8%) had mediastinal or hilar lymphadenopathy, 131 (46.5%) had pleural thickening, 77 (27.3%) had bone lesions of the ribs or thoracic vertebrae, 22 (7.8%) experienced interstitial lung disease, and 100 (35.5%) had pulmonary hypertension (Table 2). Patients exhibited one or more pulmonary manifestations. Respiratory symptoms were evident in 40.1% of the patients. The positive rate of pleural fluid and pulmonary hypertension was significantly higher in patients with respiratory symptoms than in those without the symptoms (*P* < 0.001 for both). Mild, moderate, and severe pulmonary hypertension was observed in 58 (20.6%), 31 (11%), and 11 (3.9%) patients, respectively.

**Table 2:** Characterization of chest computed tomography (CT) scan, echocardiogram, and pulmonary function tests

	ALL	No symptomes	Respiratory symptoms	*P*-value	Adjusted *P*-value^a^
N, (%)	282 (100)	169 (59.9)	113 (40.1)		
Pleural effusions, N (%)	159(56.4)	73(43.2)	86(76.1)	< 0.001	< 0.001
Unilateral, N (%)	42(14.9)	21(12.4)	21(18.6)		
Bilateral, N (%)	117(41.5)	52(30.8)	65(57.5)		
Mediastinal or hilar lymphadenopathy, N (%)	177(62.8)	99(58.6)	78(69)	0.075	0.066
Pleural thickening, N (%)	131(46.5)	80(47.3)	51(45.1)	0.716	0.753
Bone lesions of ribs or thoracic vertebra, N (%)	77(27.3)	53(31.4)	24(21.2)	0.062	0.086
Interstitial lung disease, N (%)	22(7.8)	12(7.1)	10(8.8)	0.592	0.775
Pulmonary hypertension, N (%)	100(35.5)	36(21.3)	64(56.6)	< 0.001	< 0.001
Mild, N (%)	58(20.6)	22(13)	36(31.9)		
Moderate, N (%)	31(11)	10(5.9)	21(18.6)		
Sever, N (%)	11(3.9)	4(2.4)	7(6.2)		
Heart enlargement, N (%)	114(40.4)	60(35.5)	54(47.8)	0.039	0.107
Pericardial effusions, N (%)	210(74.5)	108(63.9)	102(90.3)	< 0.001	< 0.001
Systolic dysfunction, N (%)	7(2.5)	3(1.8)	4(3.5)	0.587	0.441
Diastolic dysfunction, N (%)	108(38.3)	64(37.9)	44(38.9)	0.856	0.965
PFT					
FEV1, L	2.41 ± 0.77	2.65 ± 0.66	1.92 ± 0.77	< 0.001	< 0.001
FEV1% of predicted	83.25 ± 19.61	90.6 ± 17.38	69.71 ± 16.09	< 0.001	< 0.001
FVC, L	3.04 ± 0.9	3.29 ± 0.82	2.5 ± 0.83	< 0.001	< 0.001
FVC% of predicted	84.01 ± 21.3	90.68 ± 20.05	70.91 ± 17.47	< 0.001	< 0.001
DLCO% of predicted	64.69 ± 21.94	71.51 ± 20.3	50.33 ± 18.22	< 0.001	< 0.001
TLC% of predicted	82.07 ± 13.79	85.65 ± 11.74	74.52 ± 14.94	< 0.001	0.001
Diffuse dysfunction, N (%)	115(85.2)	75(82.4)	40(90.9)	0.193	0.149
Restrictive dysfunction, N (%)	64(47.4)	34(37.4)	30(68.2)	0.001	0.001
Obstructive dysfunction, N (%)	13(16.9)	9(9.9)	14(31.1)	0.002	0.002

*FEV*_1_ forced expiratory volume in 1 second, *FVC* forced vital capacity, *DLCO* diffusion capacity of carbon monoxide, *TLC* total lung capacity

^a^ Analyses of differences using binary logistic regression. All models were adjusted for age, sex, smoking status, history of alcohol consumption, and chronic respiratory and heart disease

### Pulmonary function test data

Diffuse dysfunction was the most frequent PFT abnormality, observed in 115 patients (85.2%) with POEMS (Table 2). Other abnormal PFT results, such as restrictive dysfunction, were detected in 64 (47.4%) patients; obstructive dysfunction was observed in 13 (16.9%) patients. After adjusting for age, sex, smoking status, history of alcohol consumption, and chronic respiratory and heart diseases, patients with respiratory symptoms had lower FEV1, FEV1%, FVC, FVC%, DLCO%, and TLC% values than those without respiratory symptoms (*P* < 0.001 for FEV1, FEV1%, FVC, FVC%, and DLCO%; and *P* = 0.001 for TLC%).

### Pleural effusion

Diagnostic thoracentesis was conducted on 16 of the 159 patients with pleural effusion. Among these, 75% of the patients met light’s criteria for exudate (fluid/serum total protein ratio > 0.5, fluid/serum LDH ratio > 0.6, or pleural fluid LDH > 2/3 of the upper limit of normal serum LDH). Table 3 presents the results of the cytological and chemical analyses of the pleural effusion.

**Table 3:** Pleural effusion analyses data in 16 patients with POEMS syndrome

Pleural effusion parameter	Value
Leukocyte, 10^6 cells/L	153 (30–231)
Monocytes, %	81.1 (66.7–85)
Protein, g/L	27.8 ± 8.6
Albumin, g/L	16.6 ± 4.2
LDH, U/L	74.0 (51.3–107.0)
ADA, U/L	4.0 ± 2.2
Glucose, mmol/L	6.0 (5.4–7.1)
Rivalta test positive, N (%)	8 (2.8)
Pleural fluid to serum protein ratio > 0.5, N (%)	8 (61.5)
Pleural fluid to serum LDH ratio > 0.6, N (%)	9 (60)
Exudate, N (%)	12 (75)

*LDH *lactatedehydrogenase, *ADA *Adenosine deaminase

### Relationship between pulmonary manifestations and the severity of POEMS syndrome

Pleural effusion and pulmonary hypertension are considered prognostic factors and participate in risk stratification. Using logistic regression, patients at high risk with poor prognosis were shown to have higher pulmonary function abnormalities (*P* < 0.001 for FEV1, FEV1%, FVC, FVC%, DLCO%, and TLC%) (Table 4). Other pulmonary imaging findings, including mediastinal or hilar lymphadenopathy, pleural thickening, rib or thoracic vertebral bone lesions, and interstitial lung disease, were not associated with the risk of POEMS syndrome.

**Table 4:** Relationship between pulmonary manifestations and the severity of POEMS syndrome

	Low risk	Intermediate risk	High risk	Adjusted *P*-value^a^
Mediastinal or hilar lymphadenopathy, N (%)	28 (51.9)	55 (59.8)	94 (69.1)	0.052
Pleural thickening, N (%)	25 (46.3)	46 (50.0)	60 (44.1)	0.497
Rib or thoracic vertebral bone lesions, N (%)	20 (37.0)	24 (26.1)	33 (24.3)	0.088
Interstitial lung disease, N (%)	2 (3.7)	6 (6.5)	14 (10.3)	0.698
Heart enlargement, N (%)	10 (18.5)	35 (38.0)	69 (50.7)	0.012
Pericardial effusions, N (%)	27 (50.0)	62 (67.4)	121 (89.0)	< 0.001
Systolic dysfunction, N (%)	0 (0.0)	1 (1.1)	1 (4.4)	0.065
Diastolic dysfunction, N (%)	18 (33.3)	36 (39.1)	54 (39.7)	0.291
PFT				
FEV1, L	3.02 ± 0.50	2.51 ± 0.58	1.79 ± 0.60	< 0.001
FEV1% of predicted	95.79 ± 14.56	85.79 ± 16.79	69.89 ± 19. 07	< 0.001
FVC, L	3.69 ± 0.63	3.11 ± 0.74	2.30 ± 0.78	< 0.001
FVC% of predicted	97.08 ± 16.53	84.64 ± 20.08	72.43 ± 20.26	< 0.001
DLCO% of predicted	78.52 ± 18.96	68.04 ± 17.17	48.27 ± 20.26	< 0.001
TLC% of predicted	88.50 ± 12.11	81.61 ± 11.70	76.81 ± 15.98	< 0.001
Diffuse dysfunction, N (%)	27 (77.1)	45 (84.9)	43 (91.5)	0.013
Restrictive dysfunction, N (%)	10 (28.6)	23 (43.4)	31 (66.0)	< 0.001
Obstructive dysfunction, N (%)	3 (8.6)	7 (13.2)	13 (27.1)	0.001

*FEV*_1_ forced expiratory volume in 1 second, *FVC* forced vital capacity, *DLCO* diffusion capacity of carbon monoxide, *TLC* total lung capacity

^a^ Analyses of differences using logistic regression. All models were adjusted for age, sex, smoking status, history of alcohol consumption, and chronic respiratory and heart disease

## Discussion

Several case reports on POEMS syndrome have described patients with pulmonary hypertension, pleural effusion, and respiratory symptoms, broadening the spectrum of POEMS syndrome components [10–13]. To enhance awareness of the association between POEMS syndrome and pulmonary disease, this study described the detailed pulmonary manifestations in 282 patients with POEMS. The most common pulmonary findings were pleural effusion, mediastinal or hilar lymphadenopathy, pleural thickening, pulmonary hypertension, diffuse dysfunction, and restrictive dysfunction. Approximately 75% of patients had exudative pleural effusion. Patients with respiratory symptoms had lower values of lung function, including FEV_1_, FEV_1_%, FVC, FVC%, DLCO%, and TLC%, than those without respiratory symptoms. Furthermore, we found that patients with poorer prognosis had worse lung function.

Respiratory complaints are frequently limited because the patient’s neurological state impairs their ability to induce symptoms [2]. Respiratory symptoms were present in 40.1% of the patients in our study. In a series of 137 patients with POEMS syndrome who visited the Mayo Clinic between 1975 and 2003, 20, 10, 8, and 7% reported dyspnea, chest pain, cough, and orthopnea, respectively [14]. Survival analysis revealed that cough and respiratory muscle weakness were associated with significantly shorter survival (*P* = 0.0014 and *P* = 0.033 for cough and muscle weakness, respectively).

Patients may present to various hospital departments with different initial symptoms. Departments of hematology, general internal medicine, and neurology were those that patients with POEMS syndrome frequently visited for medical help. This disparity may stem from greater diagnostic experience and vigilance in hematology, while other departments have insufficient awareness. Patients with multisystem symptoms may visit general internal medicine due to challenges in identifying the relevant department [16]. As polyneuropathy is the most common initial symptom, many patients are first diagnosed in the neurology department [17]. Neurology publishes the most papers and case reports on POEMS syndrome [18].

Although diagnosis of POEMS syndrome is clinically challenging, appropriate application of chest and bone radiographic assessment can be helpful in detecting it. In our study, all patients underwent chest CT or radiography, which showed accurate recognition of pulmonary abnormalities in patients with POEMS syndrome. Enlarged mediastinal or bilateral hilar nodes are unique abnormalities of this disease, and were observed in 62.8% of patients in this analysis, and this percentage was higher than that reported in other studies. Lymphadenopathy was present in 26% of the patients in the Mayo Clinic series [19]. Notably, we observed a high incidence of pleural thickening (46.5%). This manifestation has not been documented in the previous reports, necessitating the investigation of pathophysiological mechanisms. Osteosclerosis is a major diagnostic criterion of POEMS syndrome. CT is sensitive in identifying sclerotic lesions in POEMS syndrome [20, 21]. Glazebrook et al. studied 24 patients with POEMS syndrome and found that each of them had bone lesions, identified using CT, and most of which were multiple and sized less than 1 cm [21]. Previous research from our center indicated that bone lesions were frequently found in the pelvis (55/140), followed by the thoracic vertebrae (30/140), ribs (19/140), and lumbar vertebrae (14/140) [22].

Pleural effusion was another common chest manifestation in 56.4% of our study population. Wang et al. constructed and confirmed a risk nomogram that estimated the 5- and 10-year overall survival in patients with POEMS syndrome, and found that pleural effusion was a poor prognostic factor [15]. A study of 96 patients with POEMS syndrome found that pleural effusions were detected in 42.7% of patients, and all pleural fluids were exudates [23]. In our study, using multivariate logistic regression, VEGF [odds ratio (OR): 2.46, *P* = 0.01], TNF-α (OR: 3.64, *P* = 0.04), and C3 (OR: 3.77, *P* = 0.02) levels were identified as the independent risk factors for pleural effusions. In contrast to Asians, the incidence of pleural effusion in the Caucasian population, as reported by the Mayo Clinic, is 3% [19]. The pleural effusion mostly comprises exudate, mainly induced by a rapid and reversible increase in microvascular permeability caused by VEGF. Additionally, insufficient intravascular colloid osmolality in patients with hypoalbuminemia may play a role.

Lung function abnormalities were observed in most patients who underwent PFTs. Patients with respiratory symptoms exhibit poorer lung function. The most common abnormality found in the PFT results was diffusion impairment, after correcting for anemia, which was present in 85.2% of the patients. Muscle weakness or a restrictive disorder compresses the lung parenchyma, resulting in a shunt, and, ultimately, diffusion impairment [14]. Our study population’s highly restrictive pattern (47.4%) may have exacerbated abnormal diffusion. The pathogenesis of POEMS syndrome is unknown; therefore, no specific factor or gene that can predict survival, exists. Risk stratification comprises certain typical clinical manifestations or laboratory test results. A study conducted at the Mayo Clinic with 2273 person-years of follow-up found that the three factors associated with high survival were younger age, albumin levels > 3.2 g/dL, and achieving complete hematologic remission [24]. In a prognostic model developed by Wang et al., which is more appropriate for Asians, risk factors for shortened survival include age > 50, pulmonary hypertension, pleural effusion, and an estimated glomerular filtration rate of < 30 ml/min/1.73 m^2^ [15]. Notably, our study found that patients with high risk had poorer lung function, which tended to predict a worse prognosis. However, abnormalities in lung function are typically reversible. A retrospective cohort study on POEMS syndrome found that patients treated with autologous peripheral blood stem cell transplantation showed significant improvements in PFT results, respiratory muscle strength, and imaging [25]. Longitudinal data demonstrated continued improvements in FVC and DLCO (during the median follow-up at 26.5 months). Therefore, the early identification and treatment of patients with abnormal lung function are beneficial for long-term prognosis.

Patients with POEMS syndrome face an elevated risk of pulmonary hypertension, classified as World Health Organization Group 5 pulmonary hypertension, with unclear multifactorial mechanisms. Patients with extravascular overload tend to develop pulmonary hypertension [26]. It is hypothesized that elevated VEGF levels stimulate endothelial cell proliferation, leading to endothelial dysfunction and overgrowth of vascular smooth muscle, which in turn causes pulmonary hypertension [10]. He et al. found that pulmonary hypertension, which affects the right ventricular systolic and diastolic functions, was related to decreased TLC and DLCO [27]. The prevalence of patients with pulmonary conditions with POEMS syndrome may be an important factor in disease progression. A retrospective review found that 48% of patients developed pulmonary hypertension within 2 years of diagnosis [14]. After a median follow-up at 32 months, Li et al. reported that although active treatment of POEMS syndrome could reverse pulmonary hypertension, the survival of patients with pulmonary hypertension was worse than that of those without pulmonary hypertension [26]. Therefore, echocardiography should be performed to assess the presence of pulmonary hypertension in all patients with confirmed POEMS syndrome.

Our study had some limitations. First, this was a retrospective cross-sectional study with no available follow-up data. We could not analyze the changes in pulmonary manifestations and prognosis of patients with POEMS syndrome. Second, this was a single-center study, resulting in a lack of widespread screening for the disease. Finally, there were gaps in the serological results. Therefore, clinicians should be aware of the need to improve the examination of this disease.

## Conclusion

Abnormal pulmonary manifestations, including pleural effusion, mediastinal or hilar lymphadenopathy, pleural thickening, bone lesions of the ribs or thoracic vertebrae, pulmonary hypertension, diffuse dysfunction, and restrictive dysfunction, are important and common features of POEMS syndrome. Patients with respiratory symptoms exhibit compromised lung function, pleural effusion, and pulmonary hypertension. Consequently, presence of respiratory symptom warrants PFTs and echocardiography. Clinicians should intensify their awareness of the association between POEMS syndrome and pulmonary disease to enhance their understanding of the syndrome for improved diagnosis and treatment. Future research could consider conducting prospective studies or using longitudinal data to track the changes in pulmonary manifestations over time to assess the prognosis of individuals with POEMS syndrome. In this study, high-risk patients had poorer lung function, which also predicted a worse prognosis. Therefore, to enhance the generalizability of these findings, future investigations might involve multi-center studies encompassing a larger and more diverse patient population. Multi-center studies would provide a more comprehensive understanding of the disease’s prevalence and manifestations.

## Data Availability

The data are available from the corresponding author on reasonable request.

## Abbreviations

POEMS: Polyneuropathy, Organomegaly, Endocrinopathy, Monoclonal gammopathy, and Skin changes
VEGF: Vascular endothelial growth factor
IL: Interleukin
TNF-α: Tumor necrosis factor-alpha
PFT: Pulmonary function test
FEV: Forced expiration volume
FVC: forced vital capacity
DLCO: Diffusing capacity of the lung for carbon monoxide
TLC: Total lung capacity
LDH: Lactate dehydrogenase
β-CTX: β-isomerized C-terminal telopeptides
CT: Computed tomography
KAP: Kappa light chain
LAP: Lambda light chain
sPAP: Systolic pulmonary artery pressure
IQR: Inter quartile range
